# Editorial: Artificial Intelligence Applications in Specialty Crops

**DOI:** 10.3389/fpls.2022.866724

**Published:** 2022-04-21

**Authors:** Sri Charan Kakarla, Zhicheng Zhu, Yiannis Ampatzidis, Spyros Fountas, Reza Ehsani, Panos Pardalos

**Affiliations:** ^1^Department of Agricultural and Biological Engineering, Southwest Florida Research and Education Center, University of Florida, IFAS, Immokalee, FL, United States; ^2^Department of Natural Resources Management and Agricultural Engineering, Agricultural University of Athens, Athens, Greece; ^3^School of Engineering, University of California, Merced, Merced, CA, United States; ^4^Department of Industrial and Systems Engineering, University of Florida, Gainesville, FL, United States

**Keywords:** machine learning and AI, unmanned aerial vehicle (UAV), remote sensing, automation and robotics, smart farming

The rapid development of remote sensing systems, mechatronics, robotics, big data analytics, and artificial intelligence (AI) has revolutionized the specialty crop industry by allowing more precise, efficient, and cost-effective management. This Research Topic “*Artificial Intelligence Applications in Specialty Crops”* has attracted 31 high quality articles that cover the state-of-the-art applications and technical development of artificial intelligence in crop production, such as nutrient management, pest and disease management, phenotyping, and yield prediction.

Understanding the cause-effect of nutrients and the crop growth is of vital importance to the efficiency of the plant tissue culture. Conventional method using complex multivariate design of experiments (DoE) may become time consuming and lead to uninterpretable results as the number of variables increases. Garcia-Perez et al. combined artificial neural networks (ANNs) and fuzzy logic to determine the factors such as mineral nutrition affecting *Bryophyllum in vitro* growth. The result showed that the proposed model can unmask the concealed interactions and generate insights in understanding the factors involved in a certain response. Hameg et al. compared performance of different methods such as DoE, artificial neural network, fuzzy logic, and genetic algorithm (GA) in identifying and optimizing the mineral combination for hardy kiwi *in vitro* micropropagation. They demonstrated that DoE can be more efficient, and ANNs combined with fuzzy logic can understand the cause-effect of the factors and responses, and ANNs-GA can predict new mineral media formulations.

Disease diagnosis by visual observation of symptoms can get complex due to the similarity of symptoms between various diseases. This complex nature can even confuse experienced personnel to misidentify a disease which can lead to further problems. This Research Topic includes the state-of-the-art machine learning techniques that address this emerging challenges. These methods can be divided into two categories. The first type of methods developed new features and image sets that may not be visible to naked eyes for classification. For example, Feng, Wu, Zhu, et al. fused three different types of spectral datasets with visible/near-infrared hyperspectral imaging (HSI), mid-infrared spectroscopy (MIR), and laser-induced breakdown spectroscopy (LIBS) to detect three different rice diseases namely leaf blight, rice blast, and rice sheath blight. Classification models were developed using support vector machine (SVM), logistic regression (LR), and convolutional neural network (CNN) models. Best results were achieved with the models based on HSI with accuracy over 93%. Feng, Wu, He, et al. used HSI combined with deep transfer learning methods to detect rice leaf diseases in four different varieties of rice. The results suggested that this method is a promising possibility for the efficient and cost-saving field detection of rice diseases among different rice varieties. Yan et al. also used HSI data of both healthy and infected cotton plants to detect *Aphis gossypii* Glover infection. The spectra, RGB images, and hyperspectral images were used for one-dimensional, two-dimensional, and three-dimensional analysis. This study developed an initial assessment for pest detection in cotton and provides new and alternative ideas for future researchers. Khalili et al. developed and evaluated several machine learning methods to predict charcoal rot disease in soybean. And the result show the gradient tree boosting (GBT) was found to be the best performing method, which obtained 96.25 and 97.33% in terms of sensitivity and specificity. The second type of methods improved existing CNN models to achieve a better performance. For example, you only look once (YOLO) is a state-of-the-art real-time object detection algorithm, and Liu and Wang developed an improved YOLO V3 algorithm to detect tomato diseases and insect pests such as early blight, gray mold, late blight, leaf mold, leaf miner, and whitefly. The experimental results showed that the proposed algorithm has a high detection accuracy of 92.39% and a shorter detection time compared with other widely used methods such as single shot detector (SSD), fast region-based convolutional neural network (Faster R-CNN), and the original Yolo V3. Boulent et al. developed a CNN algorithm to automatically detect Flavescence Dorée symptoms across various white grapevine varieties but the true positive rates varied from 98.3 to 8.3% for different varieties which underlined the need for multi-varietal training dataset to capture the diversity of Flavescence Dorée symptoms. Mrisho et al. developed a smartphone-based object detection model called Nuru to detect symptoms of cassava mosaic disease, cassava brown streak disease, and the damage caused by cassava green mites. Results showed that Nuru can achieve a higher accuracy rate (65%) than cassava experts (researchers trained on cassava pests and diseases), agricultural extension officers, and farmers. Wang and Liu developed a multiscale parallel algorithm MP-YOLOv3 based on the MobileNetv2-YOLOv3 model. The parallel detection algorithm was used to effectively improve the detection performance of multiscale tomato gray mold lesions while ensuring the real-time performance of the algorithm. The experimental results showed that the proposed algorithm can accurately and real-time detect multiscale tomato gray mold lesions in a real natural environment. Zhu et al. utilized low resolution images of disease spots on grape leaves and used super-resolution image enhancement, which were fed into a YOLOv3-SPP network developed by them for grape leaf rot detection. The results showed that the developed method has better detection accuracy than a base CNN model, and it can be effectively used for detection of grape leaf black rot. Future works can use similar techniques for detection of small targets. Gu et al. developed a random forest model to identify different severity levels of wheat Fusarium head blight disease by fusing deep convolution features extracted from AlexNet and shallow features such as the color and the texture of wheat ears. Results showed that the proposed model has a higher accuracy than that of using either of the features alone and remains robust in terms of lights, angles, image resolutions, and crop growth periods. Yang et al. developed a model based on NASNetLarge classification model and an attention mechanism that emphasizes a target region of the image for the fine-grained classification of crop disease images. The proposed model achieved the best classification performance compared with other widely used machine learning models on different datasets. Lee et al. developed an attention-based recurrent neural network to automatically locate the infect regions for plant disease classification. The proposed model led to a better overall accuracy compared with two CNN models.

Phenotyping is an important process in the grand scheme of agricultural decision making. This process involves quantification of several plant physiological factors like plant height, plant nutrients, plant architecture, crop biomass, etc. To detect and count these factors, it requires a lot of manual effort and time. Several ground-based or remote sensing technologies combined with CNN models were used to save time and efforts required from the farmers and increase the accuracy of the detection and counting. Lin and Guo used unmanned aerial vehicles (UAVs) to acquire high resolution imagery of sorghum crop and developed a U-Net CNN model to detect and count sorghum panicles. The algorithm performed the task with an accuracy of 95.5% and a root mean square error of 2.5. Farjon et al. used two different deep learning techniques: one was a multiple scale regression and the other was a regression model that counts the leaves after locating leaf center points and aggregating them. They found that both the methods outperform the existing models in a leaf counting challenge. Hobbs et al. developed a density-estimation deep learning model based on a U-net backbone to detect flowering pineapple plants in a field. This deep learning technique runs on RGB images collected using an UAV. This allowed farmers to rapidly detect and count over 1.6 million flowering plants in a field. Hampf et al. demonstrated that a high-level accuracy in detecting pathogens and animal pests using CNN models can be achieved when it is paired with a citizen-science approach. More than 78,000 geo referenced images collected using Plantix app helped them identify yield gaps and advance the field of crop loss research. Islam et al. used a deep neural network called TheLNet270v1 to automatically classify thermal images to continuously monitor the canopy surface temperature inside a greenhouse. Liu and Wang developed an optimized YOLO-Dense algorithm based on DenseNet and YOLO V3 to detect anomalies in greenhouse tomatoes. Compared with SSD, Faster R-CNN, and the original YOLOv3 network, and the proposed model achieved the best performance in tomato anomaly detection under a complex natural environment. Apart from commonly used RGB images, there are studies that use of hyperspectral and laser spectral data combined with deep learning techniques to detect and count various factors. Zhou et al. used hyperspectral imaging of 30 different wheat varieties and developed a convolutional neural network with attention (CNN-ATT) model for wheat kernel variety identification. This model was evaluated and compared with other popular machine learning models such as SVM and partial least square discrimination analysis. The result showed that the CNN-ATT model achieved a higher accuracy than other models and the combination of hyperspectral imaging and the proposed CNN-ATT model has great potential for automatic non-destructive classification of wheat kernels. Wang W. et al. showed that the combination of laser induced breakdown spectroscopy and extreme learning machine can quickly detect the presence of cadmium in rice stems, and can accurately distinguish different degree of cadmium pollution. Bacong et al. applied CNN models to predict the entire chromatographic profile of sambong leaves under different time-varying environmental and laboratory variables parameters, which reduced the labor efforts in conventional method to evaluate herbal phytochemical composition.

Another type of technology used for phenotyping detection is segmentation. Segmentation is usually performed based on various visual factors of the crops involved to assess health, predict quality, maturity, or yield of the crop. These segmentation tasks can often be time consuming if done manually. Zhou C. et al. developed an imaging processing method, Improved ResNet, for segmenting and grading broccoli head in field conditions based on a deep learning architecture and color information. They compared this method with three other approaches, GoogleNet, VggNet, and ResNet, and illustrated that the Improved ResNet algorithm yields better results. They also showed that this algorithm can be used to obtain other data such as above ground biomass, yield, and biologic rhythm. Similarly, Ilyas et al. developed a segmentation network, named Straw-Net, for the segmentation of strawberries into four classes depending upon the ripeness of the fruit. In addition, they developed a real-time attention mechanism by integrating local and global semantic features efficiently. They compared the results with other existing segmentation models and their model demonstrated enhanced performance. For segmentation of point clouds of wheat, Ghahremani et al. developed Pattern-Net that can detect, classify, and measure features directly in the 3D point clouds with sufficient accuracy compared with manual phenotyping.

Crop yield prediction is an essential task for rapid decision-making. An accurate crop yield prediction model can help farmers to decide on what to grow and when to grow. They are also key to organizing harvesting operations. Data obtained using remote sensing technologies like RGB imagery, multispectral imagery, hyperspectral imagery combined with conventional machine learning methods can be used to predict crop yields. Johansen et al. collected UAV-based RGB and multispectral images during regular intervals prior to the harvest of tomato crops and used a random forest machine learning approach to predict end of season biomass and yield. Several shape features such as plant area, border length, width, and length were used as input variables. Both the multispectral and RGB imagery produced similar results with small differences in explained variance. Apolo-Apolo et al. used UAV-based RGB imagery to generate Orthomosaic and a region-convolutional neural network was trained to detect and count the number of apple fruit on individual trees. The results showed that an R^2^ value of 0.86 is obtained when compared with an agrotechnician's numbers. With these results, they further used Google Colab to generate yield maps, which provide a visualization of the data collected. Yoosefzadeh-Najafabadi et al. collected hyperspectral imagery of soybean in two different growth stages for yield prediction. Three common machine learning algorithms, multilayer perceptron, SVM, and random forest, were evaluated for predicting soybean seed yield using hyperspectral reflectance. The results showed that the random forest algorithm combined with ensemble–stacking method had achieved the highest performance. Kasimati et al. investigated an alternative approach to predict wine grape quality by combining UAV-based multispectral images, satellite imagery, normalized difference vegetation index, and machine learning methods. This study investigated the combination of a selection of methods required for the strongest correlation with sugar content. Wang Y. et al. developed an improved EfficientDet-D0 object detection model for wheat ear counting to evaluate crop yield. To address the challenges due to the occlusion and overlap in wheat images, they used the Random-Cutout method for image augmentation and a convolutional block attention module to refine the features. Experiments showed that the proposed model can improve the accuracy of EffientDet-D0 model by 2%.

Table 1 provides a summary of this Research Topic.

**Table 1:** Summary.

**Tasks**	**Methodologies**	**Insights**	**Articles**
Understanding the cause-effect of nutrients and the crop growth	Design of experiments (DoE), artificial neural network (ANN), genetic algorithm (GA), fuzzy logic	ANN with fuzzy logic can better unmask the interaction between nutrient and crop growth	Garcia-Perez et al., Hameg et al.
Disease diagnosis	New features combined with different classifiers, such as support vector machine (SVM), logistic regression (LR), and convolutional neural network (CNN)	The images of visible/near-infrared hyperspectral imaging (HSI), mid-infrared spectroscopy (MIR), and laser-induced breakdown spectroscopy (LIBS) can help to improve the performance	Feng, Wu, Zuh, et al., Feng, We, Hu, et al., Khalili et al., Yan et al.
	CNN	YOLO network attracts most attention, and CNN combined attention mechanism shows promising result for fine-grained classification	Boulent et al., Gu et al., Lee et al., Liu and Wang, Mrisho et al., Wang and Liu, Yang et al., Zhu et al.
Phenotype detection and counting	CNN	Most popular classification, detection and segmentation algorithms are studied, such as GoogleNet, VggNet, ResNet, YOLO, and U-Net	Bacong et al., Farjon et al., Ghahremani et al.,Hampf et al., Hobbs et al., Ilyas et al., Islam et al., Liu and Wang, Lin and Guo, Wang W. et al., Zhou L. et al., Zhou C. et al.,
Yield prediction	Random forest, SVM, neural network, CNN	An essential task is to develop a cost-effective way to collect data, and most research adopts UAVs	Apolo-Apolo et al., Johansen et al., Kasimati et al., Wang Y. et al., Yoosefzadeh-Najafabadi et al.

## Author Contributions

All authors listed have made a substantial, direct, and intellectual contribution to the work and approved it for publication.

## Conflict of Interest

The authors declare that the research was conducted in the absence of any commercial or financial relationships that could be construed as a potential conflict of interest.

## Publisher's Note

All claims expressed in this article are solely those of the authors and do not necessarily represent those of their affiliated organizations, or those of the publisher, the editors and the reviewers. Any product that may be evaluated in this article, or claim that may be made by its manufacturer, is not guaranteed or endorsed by the publisher.

